# The Pursuit of Scientific Rigor and Definitional Clarity in Health Literacy Research

**DOI:** 10.3928/24748307-20240613-01

**Published:** 2024-07

**Authors:** Alexa T. McCray

Dr. Rima Rudd's research program in health literacy is wide-ranging, encompassing health communication and education in a variety of settings, including low-resource communities both in the United States and abroad. She developed and implemented health literacy courses and tools for health educators and practitioners in a variety of settings, and she worked with policymakers to address health literacy barriers to optimal health care. Not only was she one of the key founders of the field of health literacy, but she consistently contributed to the field through her commitment to effecting systemic change in how the public interacts with health information. She collaborated with other health professionals in high-stakes emergency situations, including, perhaps most notably, with Japanese public health nurses after the 2011 Fukushima nuclear accident (Goto et al., 2018). In these situations, she brought her extensive knowledge and experience to bear in working side by side with local health professionals to develop and implement educational workshops, tools, and other resources to support improved health communication efforts in times of crisis.

A consistent theme in Dr. Rudd's work is that when health literacy interventions are proposed or implemented, they must be based on sound research in the field; they must be evaluated; and the results must contribute to building a solid foundation for the field. She argued that reaching definitional clarity on the meaning of health literacy is important for the further development and success of the broader health literacy research agenda. She consistently demonstrated scientific rigor in the approach to her own work on health literacy, and she insisted on it for the entire, still evolving, field.

When I first met Dr. Rudd, she was serving as a committee member of the Institute of Medicine's (IOM's) influential 2004 report *Health Literacy: A Prescription to End Confusion* (Institute of Medicine, 2004). She was already a well-respected public health educator and health literacy expert whose research was focused on the literacy-related barriers that patients face when attempting to interact with the health care system. The IOM report addressed these barriers directly, pointing out that health literacy needed to be understood as more than just the individual's knowledge and skills, and that, in fact, multiple factors come into play, including cultural and societal factors, the educational system, and, of course, the health care system itself. When we conversed in Washington, DC, at the launch of the IOM report, I had recently completed an extensive review of health literacy research (McCray, 2005). That review discussed some of the debates that were ongoing in the then still nascent field of health literacy, including attempts by some to broaden and reconceptualize the field. Dr. Rudd's voice and advocacy at the national level continued to contribute substantially to that reconceptualization.

Shortly after we met, I transitioned from an almost 2 decade career at the National Institutes of Health to Harvard Medical School where I would continue my research in biomedical informatics. In the years since then, I have participated in Dr. Rudd's active health literacy working group, collaborated with her on a number of projects, and have followed her work with interest.

A substantial portion of her work since the seminal IOM report has focused on the evidence base for health literacy interventions. Dr. Rudd and others argued in an article on literacy and learning in health care (Wolf et al., 2009) that because the causal pathways between health literacy and health outcomes have been so poorly understood, some health literacy interventions were bound to fail. In particular, in spite of its much broader IOM definition, health literacy is still fundamentally seen as an individual's reading ability, rather than as, they suggest, an association between “a larger set of cognitive abilities and functional literacy skills” (Wolf et al., 2009, p. S276). This broader view, which they called ‘health learning capacity,’ should, they argued, inform subsequent research in health literacy.

In Dr. Rudd's 2010 perspective on improving Americans' health literacy (Rudd, 2010), she reviewed the welcome shift that is beginning to take place in health literacy research—driven partly by both the IOM report and the then recent National Action Plan to Improve Health Literacy (U.S. Department of Health and Human Services, Office of Disease Prevention and Health Promotion, 2010). This shift in the research agenda focused more directly on the examination of the health care context and the likely barriers that patients face in interacting with the health care system.

Given this shift in focus, it seemed an optimal time to reconsider the definition of the term “health literacy” itself. We collaborated on an article that reviewed existing definitions of health literacy and argued for a new and more comprehensive definition of the term (Rudd et al., 2012). A more encompassing definition would have an impact on future research in the field, would inform policy decisions, and, importantly, would also require new measurement tools for practical implementation of health literacy interventions. A 2015 viewpoint article noted that a widening definition of the field of health literacy must include a “greater commitment and shared responsibility from clinicians, institutions, and care systems” (Koh & Rudd, 2015, p. 1226], and a longer chapter in 2017 proposed that a new definition of health literacy “could serve to shift away from a sole focus on the skills of the public to include the capacity of professionals and health institutions that support access to information and the active engagement of people” (Rudd, 2017, p. 71).

One of her most recent articles called for more rigor in science and health communication (Rudd, 2022). She made the important distinction between information that is available and information that is accessible. While there is an abundance of health information readily available to the public, the high literacy demands of much of that information makes it inaccessible to large segments of the public. She noted that information that is not accessible to the lay public “cannot build knowledge, but will, instead, inhibit use of provided information and subsequent action” (Rudd, 2022, p. 1). She argued that we must be scientific in our approach to the preparation of health materials; we must be informed by the relevant literature, methods, and protocols from a range of disciplines, including health communication and health education. Findings from health literacy studies can complement the findings from these other disciplines and can contribute to a more rigorous approach to science and health communication. Her article concluded with the following call to action: “We must be aware of our audience, take the word seriously, and shape our information with rigor. We need to be scientific in our approach” (Rudd, 2022, p. 6).
